# Higher Burden of Cerebral Small Vascular Disease Predicts Major Adverse Cardiac and Cerebrovascular Events and Is Related to Abnormal Blood Pressure Variability Pattern in Hypertension Patients

**DOI:** 10.3389/fnagi.2022.824705

**Published:** 2022-03-08

**Authors:** Xiaomeng Xu, Shu Huang, YuE Zeng, Yulan Feng, Dongqi Yue, Fanxia Shen, Yang Gao, Bei Zhang, Yang Yang, Lin Gu, Yi Fu

**Affiliations:** ^1^Department of Neurology and Institute of Neurology, Ruijin Hospital, Shanghai Jiao Tong University School of Medicine, Shanghai, China; ^2^Department of Neurology, Gongli Hospital, The Second Military Medical University, Shanghai, China; ^3^Department of Neurology, RuiJin Hospital Lu Wan Branch, Shanghai Jiao Tong University School of Medicine, Shanghai, China; ^4^Department of Neurology, Minhang Hospital, Fudan University, Shanghai, China; ^5^Department of Neurology, Ruijin North Hospital, Shanghai, China; ^6^Department of Neurology, The First Hospital of Jiaxing and The Affiliated Hospital of Jiaxing University, Zhejiang, China; ^7^Department of Radiology, Ruijin Hospital, Shanghai Jiao Tong University School of Medicine, Shanghai, China; ^8^Department of Neurology, Shanghai Second People’s Hospital, Shanghai, China; ^9^Department of Rehabilitation, Shanghai Rui Jin Rehabilitation Hospital, Shanghai, China

**Keywords:** cerebral small vascular disease, hypertension, MACCE, BPV, dipper-hypertension

## Abstract

**Background and Objectives:**

The study aims to test the hypotheses that a higher burden of cerebral small vascular disease (CSVD) predicts major adverse cardiac and cerebrovascular events (MACCE) in patients with hypertension (HTN) and that abnormal blood pressure variability (BPV) pattern aggravates total CSVD burden.

**Methods:**

We retrospectively reviewed patients with HTN prospectively selected between February 2015 and February 2019 from three participating centers. Patients were included if they had HTN for over 1 year and had at least one MRI feature of CSVD. Independent predictors were found using multivariate logistic regression.

**Results:**

Among the 908 patients who finally enrolled in the study, the number of CSVD markers (OR = 1.940; 95% CI = 1.393–2.703; *P* < 0.001) independently predicted MACCE with acceptable predictive value (C-statistic = 0.730; 95% CI = 0.669–0.791; *P* < 0.001). An abnormal BPV pattern was identified as an independent risk factor for increased CSVD burden. Among them, reverse-dipper subtype demonstrated the most significant relationship (OR = 1.725; 95% CI = 1.129–2.633; *P* = 0.012).

**Conclusion:**

Total CSVD burden predicts an increased risk of composite MACCE independently. An abnormal BPV pattern is associated with a higher burden of CSVD.

## Introduction

Cerebral small vascular disease (CSVD) is one of the most prevalent cerebral vascular pathologies encountered by the middle-aged and elderly people, with a prevalence ranging from 5% in people aged over 50 to almost 100% among people aged over 90 years (Cannistraro et al., 2019). Although the symptoms of CSVD can be clinically silent, the disease is responsible for 45–50% of all-cause dementia worldwide, as well as about a quarter of ischemic strokes and most hemorrhagic strokes (Cannistraro et al., 2019; Wardlaw et al., 2019). It is also associated with unfavorable prognosis after stroke, including worsened functional and cognitive recovery (Appleton et al., 2020), increased stroke recurrence (Lau et al., 2017), and increased mortality (Song et al., 2017).

Cerebral small vascular disease is caused by abnormalities in the small perforating arterioles, capillaries, and probably venules, featured by lacunes, high-grade white matter hyperintensities (WMH), cerebral microbleed (CMB), cortical superficial siderosis (cSS), enlarged perivascular space (EPVS), and brain atrophy on brain MRI (Wardlaw et al., 2013). The most important modifiable risk factor for CSVD is hypertension (HTN) (Hilal et al., 2017; Cannistraro et al., 2019). A systolic blood pressure intervention trial (SPRINT) substudy showed that intensive blood pressure control reduced WMH volume but led to a greater loss of total brain volume (SPRINT MIND Investigators for the SPRINT Research Group, Nasrallah et al., 2019). However, the finding is controversial, as another study suggests that excessive lowering of blood pressure may aggravate CSVD due to decreased cerebral blood flow (Denker and Cohen, 2013). In contrast, blood pressure variability (BPV) also influences CSVD (Fan et al., 2020). Circadian BPV can be classified into 4 patterns based on the percentage of systolic blood pressure (SBP) decrease at nighttime: the normal dipper pattern (10–20% of SBP drop at night), extreme-dipper (>20% of SBP drop), non-dipper (<10% of SBP drop), and reverse-dipper (SBP rise at night). Among them, the reverse-dipper pattern is proven to be associated with lacunar infarction (Yan et al., 2015). Our previous study has also shown that reverse-dipper and non-dipper patterns predict leukoaraiosis (Yang et al., 2020). However, most of the previous studies focused on one or two markers of CSVD, and less attention was paid to the total burden of CSVD. In this study, we aimed to test the hypotheses that a higher CSVD burden predicts an unfavorable prognosis in HTN patients and that an abnormal BPV pattern aggravates the total CSVD burden.

## Materials and Methods

### Patients

This study retrospectively selected prospectively included patients with HTN with cerebral small vascular pathology on MRI. The cerebral small vascular pathology was defined as the presence of at least one of the following imaging markers: lacunes, EPVS, brain atrophy, WMH (defined as Fazekas ≥ 3), and cSS. Between February 2015 and February 2019, patients were selected from Ruijin Hospital affiliated with Shanghai Jiao Tong University School of Medicine, Minhang Hospital affiliated to Fudan University, and Jiaxing First Municipal Hospital. Inclusion criteria were as follows: patients who (1) aged over 18 years; (2) were diagnosed with HTN; (3) underwent at least one brain MRI including T1, T2, and DWI sequences; (4) showed at least one MRI feature of CSVD; and (5) underwent ambulatory blood pressure monitoring (ABPM) at least once. Exclusion criteria were as follows: (1) an HTN course less than 1 year; (2) unsatisfactory MRI data or an incomplete medical record; (3) MRI identified currently acute ischemic stroke or intracranial hemorrhage in the acute stage; (4) severe medical conditions that may affect follow-up and life expectancy, including severe hepatic, renal or heart failure, and malignancy. Patients were followed for 3 years by telephone interviews or face-to-face interviews in the outpatient clinic. The composite end point of major adverse cardiac and cerebrovascular events (MACCE) was used as the end point, which included all-cause mortality, any myocardial infarction, any stroke, and any revascularization procedure (Gaudino et al., 2021). Ethical approval of this study was granted by the ethics committee of the participating centers.

### Magnetic Resonance Imaging

All of the subjects underwent brain MRI in the axial T1 WI, T2 WI, FLAIR, and DWI sequences. GE Signa HD × T 3.0 T superconducting MRI system was used for MRI acquisition. In our study, all the centers used the same imaging parameters. MRIs were reviewed by two trained investigators independently (SH and XX), and a third investigator (YF) was consulted in case of disagreement.

Cerebral small vascular disease markers were defined according to the STRIVE criteria (Wardlaw et al., 2013): (1) Lacunes were defined as a round or ovoid subcortical cavity of cerebrospinal fluid signal between 3 and 15 mm in diameter on MRI; (2) EPVS was defined as fluid-filled space following the course of penetrating vessel of cerebrospinal fluid signal on all the MRI sequences, which can be either linear (when parallel to the penetrating vessel) or round or ovoid (when perpendicular to the penetrating vessel) with a diameter less than 3 mm; (3) Brain atrophy was defined as focal or global decreased brain volume indicated by enlarged sulcal or ventricular CSF spaces, which is not related to macroscopic injuries including trauma or stroke; (4) WMHs were defined as hyperintensities on T2-FLAIR in the white matter. The severity of WMHs was scored according to Fazekas scale, and moderate (Fazekas = 3–4) to severe (Fazekas = 5–6) WMHs were marked as positive findings in our study; (5) cSS was defined as linear hypointensities along the cortical gyral surface; and (6) CMBs were small hypointense lesions visible on T2*-weighted gradient-recalled echo (GRE) or susceptibility-weighted imaging (SWI) sequences, which are generally 2–5 mm in diameter, but sometimes up to 10 mm. However, GRE and SWI were not routinely performed in all of our study centers and were, therefore, not included in the study. CMBs were not evaluated in the study due to the technique limitation.

### Ambulatory Blood Pressure Monitoring

Blood pressure variability was measured using ABPM for 24 h continuously, with a measuring gap of 30 min during the daytime (6:00–22:00) and 60 min at night (22:00 to next 6:00).

The SBP, DBP, mean BP, and heart rate were measured. The examination lasted over 24 h. Mean arterial pressure was calculated as: MAP = DBP + 1/3 × (SBP - DBP). In addition, the nocturnal blood pressure declines rate (NBPDR) was calculated as NBPDR = (Diurnal MAP - Nocturnal MAP)/Diurnal MAP. The BPV pattern was identified according to NBPDR: dipper pattern was defined as NBPDR between 10 and 20%, the extreme-dipper pattern was defined as NBPDR ≥ 20%, the non-dipper pattern was defined as NBPDR between 0 and 10%, and the reverse-dipper pattern was defined as NBPDR < 0 (Yan et al., 2015).

### Statistics

The continuous data [age, cholesterol, triglycerides, low-density lipoprotein (LDL), and high-density lipoprotein (HDL)] were transformed into dichotomous variables with cutoff values shown in Table 1. Categorical data were expressed as frequencies and percentages and were compared using chi-square tests or Fisher’s exact tests, when appropriate. Variables with *P* < 0.1 and variables relevant to our research (lacunes, EPVS, brain atrophy, WMH, cSS, and BPV subtype) (Yang et al., 2020; Xu et al., 2021) were included in a subsequent multivariate logistic regression to identify independent predictors. When exploring predictors for MACCE, each CSVD marker and total CSVD marker numbers were included in two distinct models to avoid collinearity because they were mutually closely correlated. The discriminative capability was compared using the area under the receiver operating characteristic (ROC) curve (AUC), and the optimal cutoff value was determined using the Youden index. All the analyses were performed using SPSS 24.0 (IBM, Armonk, NY, United States). A two-sided *P* < 0.05 was considered statistically significant.

**TABLE 1:** Demographics and clinical characteristics.

	MACCE = 0	MACCE = 1	*P*	95% CI	
	*n* = 856	*n* = 52			
Age (≥70), *n* (%)	404 (47.2%)	39 (75.0%)	<0.001	1.767	6.377
Sex, *n* (%)			0.056	0.316	1.021
Female, *n* (%)	413 (48.52%)	18 (34.6%)			
Male, *n* (%)	443 (51.8%)	34 (65.4%)			
Smoking, *n* (%)	188 (22.0%)	16 (30.8%)	0.144	0.854	2.896
Drinking, *n* (%)	126 (14.9%)	10 (19.2%)	0.395	0.666	2.785
**Previous history**					
Atrial fibrillation, *n* (%)	35 (4.1%)	5 (9.6%)	0.059	0.935	6.663
Diabetes mellitus, *n* (%)	305 (35.6%)	23 (44.2%)	0.21	0.814	2.521
Previous stroke, *n* (%)	41 (4.8%)	4 (7.7%)	0.349	0.57	4.816
Carotid plaque, *n* (%)	574 (67.1%)	46 (88.5%)	0.001	1.59	8.924
**Medication**					
Antiplatelet, *n* (%)	101 (11.8%)	14 (26.9%)	0.001	1.442	5.26
Anticoagulants, *n* (%)	12 (1.4%)	2 (3.8%)	0.165	0.613	12.913
Statin, *n* (%)	104 (12.1%)	8 (15.4%)	0.491	0.602	2.87
**Dyslipidemia**					
Cholesterol ≥ 6.2 mmol/L, *n* (%)	49 (5.8%)	2 (3.9%)	0.567	0.155	2.786
Triglycerides ≥ 5.7 mmol/L, *n* (%)	29 (3.4%)	1 (19%)	0.555	0.073	4.122
LDL ≥ 4.14 mmol/L, *n* (%)	49 (5.8%)	4 (7.7%)	0.579	0.467	3.893
HDL ≤ 1 mmolL, *n* (%)	316 (37.5%)	19 (36.5%)	0.886	0.536	1.714
**Imaging characteristics**					
Lacunes, *n* (%)	512 (59.8%)	34 (65.4%)	0.426	0.705	2.284
EPVS, *n* (%)	547 (63.9%)	40 (76.9%)	0.057	0.973	3.643
Brain atrophy, *n* (%)	363 (42.4%)	41 (78.8%)	<0.001	2.567	9.984
WMH (Fazekas ≥ 3), *n* (%)	397 (46.4%)	40 (76.9%)	<0.001	1.994	7.449
cSS, *n* (%)	10 (1.2%)	2 (3.8%)	0.101	0.722	15.86
Total number of CSVD markers			<0.001		
1	307 (35.9%)	2 (3.8%)			
2	218 (25.5%)	12 (23.1%)			
3	241 (28.2%)	22 (42.3%)			
4	87 (10.2%)	15 (28.8%)			
5	3 (0.4%)	1 (1.9%)			
**BPV subtype**			**0.052**		
Dipper	229 (26.8%)	6 (11.5%)			
Non-dipper	393 (45.9%)	28 (53.8%)			
Reverse-dipper	217 (25.4%)	18 (34.6%)			
Extreme-dipper	17 (2.0%)	0 (0.0%)			

*CSVD, cerebral small vascular disease; MACCE, major adverse cardiac and cerebrovascular events; WMH, white matter hyperintensities; EPVS, enlarged perivascular space; cSS, cortical superficial siderosis; BPV, blood pressure variability; LDL, low-density lipoprotein; HDL, high-density lipoprotein; CI, confidence interval.*

### Standard Protocol Approvals, Registrations, and Patient Consents

The study was approved by the ethics committee of all the participating centers. Informed consent was exempted due to the purely observational nature of the study.

### Data Availability Statement

All of the de-identified data used in this study are available upon reasonable request from the corresponding author.

## Results

### Demographics and Clinical Characteristics

**FIGURE 1:**
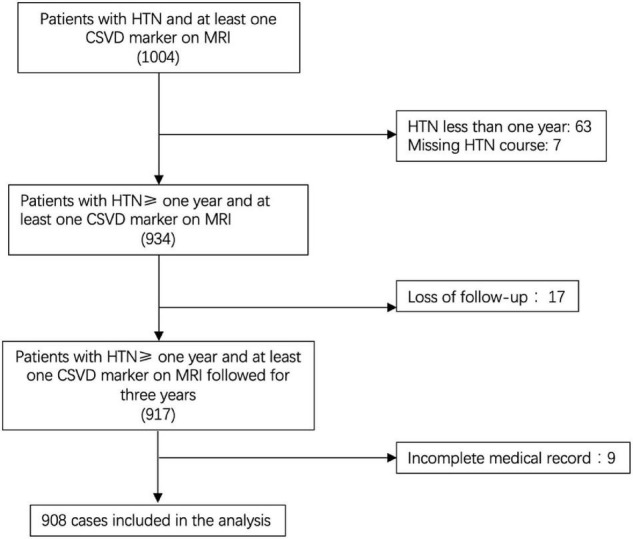
Flowchart of patient inclusion. CSVD, cerebral small vascular disease; HTN, hypertension.

The study included a total of 1,004 patients according to the pre-established inclusion criteria. Among them, 63 with HTN less than 1 year were excluded and seven without HTN course data were excluded. In the remaining 934 patients, 17 were excluded due to loss of follow-up, and nine were excluded due to an incomplete medical record. Among the 908 patients who finally enrolled in the analysis (Figure 1), the mean age was 69.06 years (SD = 12.23), 477 were men (52.5%), 431 were women (47.5%), and the average course of HTN was 14.48 years (SD = 10.70). At follow-up, MACCE occurred in 52 patients (5.73%), and among them, 22 died at the 3-year follow-up (2.42%).

### Imaging Markers of Cerebral Small Vascular Disease Predicts Major Adverse Cardiac and Cerebrovascular Event

Our univariate analysis revealed that age over 70 years, male sex, atrial fibrillation, carotid plague, currently on antiplatelet therapy, MRI markers of CSVD, and HTN subtype were significantly associated with MACCE before adjustment (Table 1). Detailed ABPM data are shown in Supplementary Table 1. Nighttime BP fall rate significantly differed between patients with and without MACCE during follow-up (Supplementary Table 1, *P* = 0.002), suggesting that non-dipper HTN subtype may be related to MACCE.

We used two multivariate logistic regression models to avoid collinearity, since the total number of CSVD markers was theoretically dependent on the presence of each CSVD imaging marker. Nighttime BP fall rate was not included in the regression model due to its collinearity with HTN subtype. As a result, male (model 1: OR = 1.896; 95% CI = 1.03–3.488; *P* = 0.040; model 2: OR = 1.857; 95% CI = 1.012–3.409; *P* = 0.046), current antiplatelet therapy (model 1: OR = 2.093; 95% CI = 1.055–4.153; *P* = 0.035; model 2: OR = 2.113; 95% CI = 1.068–4.179; *P* = 0.032), brain atrophy (model 1: OR = 2.838; 95% CI = 1.271–6.338; *P* = 0.011), and the number of CSVD markers (model 2: OR = 1.940; 95% CI = 1.393–2.703; *P* < 0.001) independently predicted MACCE in follow-up (Table 2). The C-statistic was used to evaluate the prognostic value of the presence of each CSVD imaging marker and the total number of CSVD markers (Supplementary Figure 1). Table 3 shows the AUC, which showed the highest C-statistic in the number of CSVD markers (AUC = 0.730; 95% CI = 0.669–0.791; *P* < 0.001). The Youden’s index indicated that the presence of 3 or more CSVD markers predicted MACCE with highest sensitivity and specificity, as the cutoff value was 2.5 (Youden’s index = 0.344; sensitivity = 0.731; specificity = 0.613; Table 4).

**TABLE 2:** Multivariate logistic regression analysis for risk factors of MACCE.

	*P*	OR	95%CI	
**Model 1**				
Age ≥ 70	0.469	1.333	0.612	2.902
Male	0.040	1.896	1.03	3.488
Atrial fibrillation	0.475	1.465	0.514	4.170
Carotid plaque	0.193	1.844	0.734	4.634
Antiplatelet	0.035	2.093	1.055	4.153
Lacunes	0.189	1.573	0.801	3.089
EPVS	0.374	1.423	0.654	3.097
Brain atrophy	0.011	2.838	1.271	6.338
cSS	0.412	1.982	0.386	10.168
WMH (Fazekas ≥ 3)	0.056	2.009	0.982	4.112
BPV subtype (relative to dipper)	0.313			
Non-dipper	0.061	2.427	0.960	6.134
Reverse-dipper	0.115	2.191	0.827	5.805
Extreme-dipper	0.999	0.000	0.000	/
**Model 2**				
Age ≥ 70	0.207	1.593	0.773	3.282
Male	0.046	1.857	1.012	3.409
Atrial fibrillation	0.447	1.500	0.528	4.261
Carotid plaque	0.193	1.840	0.735	4.609
Antiplatelet	0.032	2.113	1.068	4.179
Number of CSVD markers	0.000	1.940	1.393	2.703
BPV subtype (relative to dipper)	0.318			
Non-dipper	0.062	2.413	0.956	6.091
Reverse-dipper	0.114	2.197	0.829	5.820
Extreme-dipper	0.999	0.000	0.000	/

*CSVD, cerebral small vascular disease; WMH, white matter hyperintensities; EPVS, enlarged perivascular space; cSS, cortical superficial siderosis; BPV, blood pressure variability; OR, odds ratio; CI, confidence interval.*

**TABLE 3:** Evaluation of the predictive value of CSVD markers using C-statistic.

	AUC	Standard error	*P*	95% CI	
Lacunes	0.528	0.041	0.499	0.448	0.608
EPVS	0.565	0.039	0.115	0.489	0.641
Brain atrophy	0.682	0.035	<*0*.*001*	0.613	0.751
cSS	0.513	0.042	0.746	0.431	0.596
WMH (Fazekas ł 3)	0.653	0.036	<*0*.*001*	0.581	0.724
Number of CSVD markers	0.73	0.031	<*0*.*001*	0.669	0.791

*CSVD, cerebral small vascular disease; WMH, white matter hyperintensities; EPVS, enlarged perivascular space; cSS, cortical superficial siderosis; AUC, area under curve; CI, confidence interval.*

**TABLE 4:** Optimal cutoff value of the number of cerebral small vascular disease (CSVD) markers predicting major adverse cardiac and cerebrovascular events (MACCE).

Number of SVD markers	Sensitivity	1 – specificity	Youden’s index
0	1	1	0
1.5	0.962	0.641	0.321
2.5	0.731	0.387	0.344
3.5	0.308	0.105	0.203
4.5	0.019	0.004	0.015
6	0	0	0

### Hypertension Subtype Correlates to Cerebral Small Vascular Disease Burden

We subsequently analyzed potential predictors of increased CSVD burden based on the total number of CSVD markers. The univariate analysis revealed that age over 70 years, smoking, drinking, atrial fibrillation, diabetes mellitus, previous stroke, carotid plaque, antiplatelet therapy, and HTN subtype were associated with increased CSVD burden, indicated by the number of CSVD markers ≥ 3 (Table 5). Among them, age over 70 years (OR = 5.492; 95% CI = 3.918–7.699; *P* < 0.001), previous stroke (OR = 2.818; 95% CI = 1.372–5.789; *P* = 0.005), carotid plaque (OR = 1.855; 95% CI = 1.281–2.697; *P* = 0.001), and HTN subtype (relative to dipper) stood out as independent risk factors for increased CSVD burden after adjustment for confounding variates (Table 6). Among all the HTN subtypes, reverse-dipper subtype was most significantly related to increased CSVD burden (OR = 1.725; 95% CI = 1.129–2.633; *P* = 0.012), whereas non-dipper subtype indicated a suggestive trend (OR = 1.387; 95% CI = 0.949–2.028; *P* = 0.091).

**TABLE 5:** Univariate analysis of predictors of the number of small vascular disease (SVD) markers ≥ 3.

	Number of SVD markers ≤ 2 *n* = 539	Number of SVD markers ≥ 3 *n* = 369	*P*	CI	
Age (≥70), *n* (%)			<0.001	4.802	8.692
<69, *n* (%)	371 (68.8%)	94 (25.5%)			
≥70, *n* (%)	168 (31.2%)	275 (74.5%)			
**Sex, n (%)**					
Female, *n* (%)	255 (47.3%)	176 (47.7%)	0.909	0.779	1.324
Male, *n* (%)	284 (52.7%)	193 (52.3%)			
Smoking, *n* (%)	135 (25.1%)	69 (18.8%)	0.026	0.499	0.958
Drinking, *n* (%)	96 (18.0%)	40 (10.9%)	0.004	0.376	0.83
**Previous history**					
Atrial fibrillation, *n* (%)	16 (3.0%)	24 (6.5%)	0.011	1.191	4.343
Diabetes mellitus, *n* (%)	179 (33.2%)	149 (40.4%)	0.027	1.035	1.792
Previous stroke, *n* (%)	19 (3.5%)	26 (7.0%)	0.016	1.131	3.807
Carotid plaque, *n* (%)	313 (58.1%)	307 (83.2%)	<0.001	2.591	4.934
**Medication**					
Antiplatelet, *n* (%)	53 (9.8%)	62 (16.8%)	0.002	1.249	2.745
Anticoagulants, *n* (%)	6 (1.1%)	8 (2.2%)	0.205	0.677	5.721
Statin, *n* (%)	64 (11.9%)	48 (13.0%)	0.610	0.744	1.656
**Dyslipidemia**					
Cholesterol ≥ 6.2 mmol/L, *n* (%)	33 (6.2%)	18 (5.0%)	0.430	0.437	1.424
Triglycerides ≥ 5.7 mmol/L, *n* (%)	20 (3.8%)	10 (2.8%)	0.412	0.335	1.568
LDL ≥ 4.14 mmol/L, *n* (%)	30 (5.6%)	23 (6.4%)	0.657	0.648	1.988
HDL ≤ 1 mmol L, *n* (%)	194 (36.5%)	141 (39.0%)	0.451	0.844	1.464
**BPV subtype**			**<0.001**		
Dipper	162 (30.1%)	73 (19.8%)			
Non-dipper	249 (46.2%)	172 (46.6%)			
Reverse-dipper	112 (20.8%)	123 (33.3%)			
Extreme-dipper	16 (3.0%)	1 (0.3%)			

*BPV, blood pressure variability; LDL, low-density lipoprotein; HDL, high-density lipoprotein; CI, confidence interval.*

**TABLE 6:** Multivariate analysis of predictors of the number of SVD markers ≥ 3.

	*P*	OR	95% CI	
Age ≥ 70	<0.001	5.492	3.918	7.699
Smoking	0.117	1.447	0.912	2.298
Drinking	0.447	0.811	0.472	1.393
Atrial fibrillation	0.362	1.411	0.672	2.962
Diabetes mellitus	0.661	1.073	0.783	1.472
Previous stroke	0.005	2.818	1.372	5.789
Carotid plaque	0.001	1.855	1.281	2.687
Antiplatelet	0.103	1.467	0.926	2.325
BPV subtype (relative to dipper)	0.018			
Non-dipper	0.091	1.387	0.949	2.028
Reverse-dipper	0.012	1.725	1.129	2.633
Extreme-dipper	0.110	0.174	0.021	1.480

*BPV, blood pressure variability; OR, odds ratio; CI, confidence interval.*

## Discussion

Our study has two major findings: first, total CSVD burden, as represented by the number of CSVD markers, independently predicts poor prognosis, as indicated by the composite end point of MACCE; and second, abnormal BPV pattern, specifically the reverse-dipper subtype, is associated with a higher burden of CSVD.

A major adverse cardiac and cerebrovascular event is an established composite end point of cardio- and cerebral vascular events and all-cause death. Previous studies have identified the association between CSVD and the occurrence, recurrence, and prognosis of cerebral infarction, intracranial hemorrhage, and myocardial infarction (Ikram et al., 2008; Song et al., 2017; Park et al., 2019; Guey et al., 2021; Rodrigues et al., 2021). For example, a sub-analysis of the prevention of cardiovascular events in Asian patients with ischemic stroke at high risk of cerebral hemorrhage (PICASSO) study found that CSVD burden presented by the Fazekas scale score of WHM correlated with an increased risk of recurrent stroke and major vascular events. Notably, as the PICASSO study prospectively enrolled patients, the outcome was recurrent, rather than first-ever, stroke and major vascular events (Park et al., 2019). Besides, an earlier study demonstrated that the total CSVD score, which represents the overall CSVD burden, was associated with increased mortality in long-term follow-up of patients with acute stroke or TIA, with a median follow-up of 3.8 years (Song et al., 2017). A recent study further supported that CSVD is associated with 1-year death and dependence in patients with ICH (Rodrigues et al., 2021). In the field of cardiology, a previous cross-sectional analysis of the Rotterdam study proved that unrecognized myocardial infarction was related to dementia and CSVD (Ikram et al., 2008). However, the mechanism by which CSVD increases MACCE is not clear. One possible explanation is that cardiovascular and cerebrovascular risk factors share similar risk factors and underlying vascular pathogenesis. In addition, reduced white matter blood flow, vascular reserve, and fragility of arteriolar bed may contribute to increased risk of stroke (Park et al., 2019). One of our study’s strengths is that the hypertensive population has less confounding baseline cerebral vascular damages, which may bias functional outcome and mortality.

Our second finding, BPV promotes CSVD, was also consistent with previous reports. A recent study based on the Rotterdam study demonstrated that increased visit-to-visit BPV led to subclinical brain microstructure alterations including CSVD (Ma et al., 2020). In contrast, our previous study found that abnormal blood pressure circadian rhythms correlated with cerebral infarction and leukoaraiosis (Yang et al., 2020). However, how abnormal BPV promotes cerebral structure remains to be clarified (Iadecola and Parikh, 2020). It is possible that abnormal blood variation patterns influenced the pulsation patterns of blood and dampened small arteries and interfered with endothelial functions, finally causing vascular events (Ma et al., 2020). Prospective studies in the future are necessary to further elucidate the potential mechanism (Yan et al., 2015).

Our findings failed to demonstrate a significant association between BPV and MACCE. Although a potential tendency toward significance was detected in unadjusted comparison, the tendency disappeared after adjustment for other risk factors. One possible explanation is that BPV correlates with other risk factors in the logistic regression model. To support the idea, we subsequently demonstrated that BPV was highly associated with CSVD burden, which independently predicts MACCE. Therefore, an abnormal BPV pattern may aggravate small vessel damage, which indirectly affects the outcome.

The absence of SWI and T2* sequences was a major limitation of the evidence. Therefore, data of CMBs and well-cited CSVD scales (Staals et al., 2014; Charidimou et al., 2016; Lau et al., 2017) are not applicable in the study. The overall CSVD burden was calculated using the total number of CSVD markers. Although not as widely used as the established CSVD scales, the C-statistic showed a relatively acceptable predictive value (AUC = 0.730). In addition, due to the lack of SWI and T2* sequences, cSS was evaluated using T2-Flair. However, T2-Flair is not the ideal sequence for cSS, and SWI can detect cSS with a higher sensitivity (Haller et al., 2021). A recent study showed SWI can detect more cSS than 3D-Flair (Umino et al., 2021). However, the basic sequences used in our study are cost-effective for general screening of potential cerebral vascular diseases, rather than a further evaluation of patients with cerebral vascular diseases in the neurology department. Since the progress of CSVD is a relatively chronic process, another limitation of our study is the relatively short follow-up, which may underestimate the incidence of MACCE, although we have already found a statistical association between CSVD burden and MACCE. In addition, we cannot exclude patients with WMHs caused by other less common etiologies such as inflammation or metabolism. A final limitation is the retrospective nature of the study. For example, we identified antiplatelets as a predictor of MACCE, probably because physicians tend to prescribe antiplatelets to patients with more CVD risk factors. In the future, prospective studies with longer follow-up may address the limitations and confirm the findings.

## Data Availability Statement

The original contributions presented in the study are included in the article/Supplementary Material, further inquiries can be directed to the corresponding authors.

## Author Contributions

XX, LG, and YFu: drafting and revision of the manuscript for content, including medical writing for content, study concept or design, and analysis or interpretation of data. SH: drafting and revision of the manuscript for content, including medical writing for content, and analysis or interpretation of data. YZ: study concept or design and analysis or interpretation of data. YFe, DY, FS, YG, BZ, and YY: major role in the acquisition of data, drafting and revision of the manuscript for content, and including medical writing for content. All authors contributed to the article and approved the submitted version.

## Conflict of Interest

The authors declare that the research was conducted in the absence of any commercial or financial relationships that could be construed as a potential conflict of interest. The handling editor G-YY and the reviewer BB declared a shared parent affiliation with several of the authors XX, YZ, DY, FS, BZ, LG, and YFu at the time of the review.

## Publisher’s Note

All claims expressed in this article are solely those of the authors and do not necessarily represent those of their affiliated organizations, or those of the publisher, the editors and the reviewers. Any product that may be evaluated in this article, or claim that may be made by its manufacturer, is not guaranteed or endorsed by the publisher.

## Supplementary Material

Supplementary Figure 1 The ROC curves of each and the total number of CSVD markers.
