# Supplementary figures and images for: Higher Burden of Cerebral Small Vascular Disease Predicts Major Adverse Cardiac and Cerebrovascular Events and Is Related to Abnormal Blood Pressure Variability Pattern in Hypertension Patients
Source: Front Aging Neurosci. 2022 Mar 8;14:824705. doi: 10.3389/fnagi.2022.824705 (PMC8959137; doi:10.3389/fnagi.2022.824705)

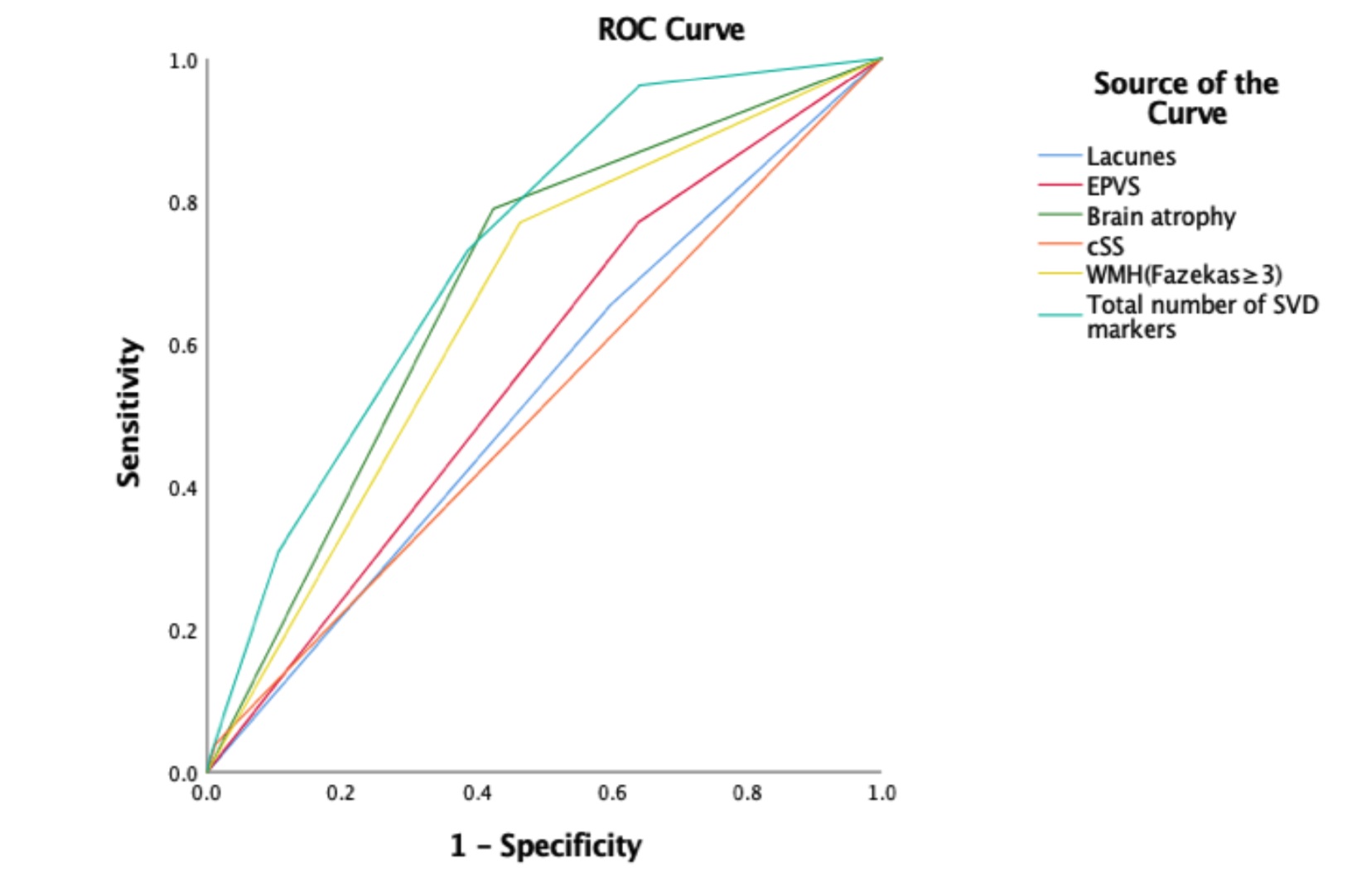

Supplement: Supplementary Figure 1 — The ROC curves of each and the total number of CSVD markers. [file Image_1.jpeg]
